# Frontiers of Sodium MRI Revisited: From Cartilage to Brain Imaging

**DOI:** 10.1002/jmri.27326

**Published:** 2020-08-26

**Authors:** Olgica Zaric, Vladimir Juras, Pavol Szomolanyi, Markus Schreiner, Marcus Raudner, Chiara Giraudo, Siegfried Trattnig

**Affiliations:** ^1^ Institute for Clinical Molecular MRI in the Musculoskeletal System Karl Landsteiner Society Vienna Austria; ^2^ High‐Field MR Center, Department of Biomedical Imaging and Image‐guided Therapy Medical University of Vienna Vienna Austria; ^3^ Department of Imaging Methods, Institute of Measurement Science Slovak Academy of Sciences Bratislava Slovakia; ^4^ Deartment of Orthopaedics and Trauma Surgery Medical University of Vienna Vienna Austria; ^5^ Radiology Institute, Department of Medicine DIMED Padova University Via Giustiniani 2 Padova Italy; ^6^ Christian Doppler Laboratory for Clinical Molecular MRI Christian Doppler Forschungsgesellschaft Vienna Austria

**Keywords:** sodium, MRI, technical developments, clinical applications

## Abstract

Sodium magnetic resonance imaging (^23^Na‐MRI) is a highly promising imaging modality that offers the possibility to noninvasively quantify sodium content in the tissue, one of the most relevant parameters for biochemical investigations. Despite its great potential, due to the intrinsically low signal‐to‐noise ratio (SNR) of sodium imaging generated by low in vivo sodium concentrations, low gyromagnetic ratio, and substantially shorter relaxation times than for proton (^1^H) imaging, ^23^Na‐MRI is extremely challenging. In this article, we aim to provide a comprehensive overview of the literature that has been published in the last 10–15 years and which has demonstrated different technical designs for a range of ^23^Na‐MRI methods applicable for disease diagnoses and treatment efficacy evaluations. Currently, a wider use of 3.0T and 7.0T systems provide imaging with the expected increase in SNR and, consequently, an increased image resolution and a reduced scanning time. A great interest in translational research has enlarged the field of sodium MRI applications to almost all parts of the body: articular cartilage tendons, spine, heart, breast, muscle, kidney, and brain, etc., and several pathological conditions, such as tumors, neurological and degenerative diseases, and others. The quantitative parameter, tissue sodium concentration, which reflects changes in intracellular sodium concentration, extracellular sodium concentration, and intra–/extracellular volume fractions is becoming acknowledged as a reliable biomarker. Although the great potential of this technique is evident, there must be steady technical development for ^23^Na‐MRI to become a standard imaging tool. The future role of sodium imaging is not to be considered as an alternative to ^1^H MRI, but to provide early, diagnostically valuable information about altered metabolism or tissue function associated with disease genesis and progression.

**Level of Evidence:**

1

**Technical Efficacy Stage:**

1

IN VIVO SODIUM MAGNETIC RESONANCE IMAGING (^23^Na‐MRI) is an advanced imaging modality that offers noninvasive metabolic imaging for early and accurate diagnosis, characterization, and treatment efficacy evaluations of several diseases.

The ^23^Na ion is one of the most important electrolytes in the human body and it plays a crucial role in osmoregulation and physiology of the cell.^1^ A large sodium concentration gradient is required for appropriate function of the cells, and intracellular sodium concentration (ISC) is one‐tenth of that in the extracellular space (ESC) (10–15 vs. 100–150 mmol/L). In healthy tissue, the large concentration gradient between the cells and the extracellular space is maintained primarily by the energy‐dependent sodium–potassium pump (Na^+^/K^+^‐ATPase). If the cell membrane or energy metabolism is destroyed, it can drive an impairment of the sodium–potassium pump, which will further increase intracellular sodium concentration and induce cell malfunction and, eventually, cell death.^2^

An in vivo ^23^Na MRI can noninvasively provide valuable information on cell metabolism; however, the technique is extremely challenging. ^23^Na has a spin of 3/2 and belongs to the group of quadrupole nuclei. The sensitivity of nuclear magnetic resonance (NMR) experiments is given by signal‐to‐noise‐ratio (SNR) and, for ^23^Na, it is 9.2% that of proton (^1^H) sensitivity. There are three major factors that limit the SNR of sodium imaging: the concentration of the ^23^Na ion in vivo is low and is in the range from 15 mM (muscle 15–30 mM) to 350 mM (articular cartilage 250–350 mM); relaxation times of sodium are about two orders of magnitude smaller than those of protons (T_1_: 15–55 msec, T_2short_: 0.5–2.5 msec, and T_2long_: 10–65 msec), and the gyromagnetic ratio of sodium (γ_Na_ = 11.26 MHz/T) is approximately four times lower than that for hydrogen (γ_H_ = 42.57 MHz/T).^3^ Fortunately, most of these limitations were abolished by the introduction of 3.0T (high‐field) and ≥7.0T (ultrahigh‐field) scanners and an essential SNR increase was achieved (SNR ~ B_0_^1.65^).^4^ The modern MRI systems are currently equipped with strong gradients and sophisticated electronics, multiarray coils, and fast (non‐Cartesian) sequences that allow further SNR enhancement of ^23^Na‐MRI.^5^

The purpose of this article is to provide readers with an overview of the current literature, including methodological improvements in the ^23^Na‐MRI technique and its application in preclinical and clinical investigations at 3.0T and 7.0T. Lately, several review articles that cover some important aspects of the technique have been published, including biomedical applications for sodium imaging, evaluations of cartilage repair techniques and osteoarthritis, sodium imaging of the heart and the brain, ^23^Na‐MRI radiofrequency (RF) systems for brain and musculoskeletal or body imaging, and quantitative sodium imaging, etc.^3^, ^6^, ^7^, ^8^, ^9^, ^10^, ^11^, ^12^ Considering that the majority of previously published review articles were focused on a single organ or disease, we attempt here to summarize a significant amount of new publications since the last comprehensive sodium review article appeared. In this work, we aim to provide a review of recent ^23^Na‐MRI findings when applied in nearly all parts of the body and revisit the frontiers of sodium imaging in modern medicine.

## Imaging Sequences

^23^Na in tissue has a biexponential relaxation behavior, which means that most of the ^23^Na signal is lost within a few milliseconds. The rapid signal loss renders quantitative imaging challenging. The short T_1_ relaxation time facilitates a short repetition time (TR) and fast averaging, which can partially compensate for the low intrinsic SNR. Imaging with pulse sequences designed in a way that enables measurements with a very short echo time (TE, ~1 msec), such as an ultrashort echo time (UTE) sequence, may overcome the challenge of the very short T_2_ of sodium nuclei. On the other hand, a short TE will substantially limit the duration of the signal readout and cause image blurring and a decrease in SNR. However, UTE acquisition methods are generally slow compared to echo‐sampled MRI and the sampling of *k*‐space with high efficacy is an important factor in achieving high SNR and resolution in an acceptable scan time.

The development of non‐Cartesian sequences for sodium imaging that aim to maximize the efficiency of *k*‐space sampling started in early 1990 and continues today. Hilal et al in 1992 demonstrated the feasibility of a three‐dimensional radial projection (3DRPI) acquisition method in which the center of *k*‐space is densely sampled, while the edges of *k*‐space are undersampled.^13^ Almost a decade later, Nagel et al developed a pulse sequence, called the density‐adapted three‐dimensional radial projection reconstruction pulse sequence (DA‐3DPR) that was designed on the basis of a conventional 3DPR sequence. The sequence was modified such that the sampling density in the outer part is kept constant, while an inner sphere of *k‐*space is sampled with no density adaptation. This approach enabled substantially improved image quality and an increase in SNR.^14^

In parallel, Boada et al developed the twisted projection imaging (TPI) sampling scheme.^15^ This method replaced radial linear gradients with time‐varying gradients with a short radial component to move promptly away from the center of *k‐*space, followed by a time‐inconstant gradient that dismissed a 3D spiral trajectory.^15^, ^16^ TPI uses spokes up to a threshold *k‐*space value, at which point the trajectory transits into a 3D spiral‐like trajectory.^16^ This sequence was shown to be the most favorable for in vivo quantitative sodium measurements in brain.^17^ Shortly after, Boada's group developed a sequence called the acquisition‐weighted stack of spirals (AWSOS) sequence, which attempts to decrease excitation and acquisition delay by introducing a variable slice‐encoding, and separating slice thickness from in‐plane resolution to lower the number of slice‐encoding steps, while at the same time, using a spiral readout to increase the efficiency of in‐plane acquisition.^18^ In addition to the above‐mentioned sequences, other *k‐*space trajectories, ie, employing distributed spiral trajectories, such as SPRITE (Single‐Point Ramped Imaging with T1‐Enhancement) and FLORET (Fermat Looped, Orthogonally Encoded Trajectories), have been proposed and used for ^23^Na‐MRI.^19^, ^20^

The necessity to increase SNR and image resolution of ^23^Na‐MRI was accompanied by the needs of researchers and clinicians for selective measurements of intracellular sodium changes. Their goal was to develop a method that could provide more specific information and an image biomarker of compromised ionic homeostasis. Biexponential relaxation, typical of sodium nuclei in slow motion in the intracellular space, also observable in the extracellular space, can pass through a state of multiple quantum coherences (MQC).^21^ MQ filtering techniques are essentially sensitive to changes in intracellular sodium concentration, and therefore, these techniques are ideally suited for the noninvasive, in vivo observations of ISC level changes. Tsang et al confirmed the possibility of double quantum‐magic angle (DQ‐MA) signal generation from the human brain.^22^ Initial experiments, performed with the highest nominal isotropic resolution of (8.4 mm)^3^ and 48 minutes scanning time, demonstrated the presence of sodium nuclei in ordered environments. Later on, the DQ‐MA method was used to visualize the sodium signal that originates from anisotropic structures, such as muscle fibers.^23^

However, the main drawback of the MQ techniques is their low SNR. Using a train of three RF pulses (with the corresponding phase cycling), Hancu et al demonstrated that 3D triple‐quantum‐filtered (TQF) sodium images of the human brain can be acquired at moderate field strengths (3.0T) with examination times acceptable for clinical applications.^24^ Tsang et al, however, demonstrated the utilization of sufficiently long RF pulses and a reduced TR that might lead to further SNR enhancement for TQF images.^25^

Essentially, low SNR was not the only issue with TQF sequences; they entail a lower power deposition and they are still prone to image artifacts caused by off‐resonances. Therefore, development continued and a method called biexponentially weighted (BW) ^23^Na‐MRI was developed. The initial results published were very promising, and demonstrated a three times higher image SNR obtained by BW ^23^Na compared to the six‐step phase‐cycling TQF MRI.^26^ For the excitation and detection of MQC, a three‐pulse preparation is applied during the pulse train, and two images are generated: a spin‐density‐weighted image (SDW), and a single‐quantum‐filtered image (SQF). The BW image is derived by subtracting the SQF image from the SDW image and shows the signal from sodium ions with biexponential relaxation. Nagel et al proposed a relaxation‐based (RW) method as a possibility to differentiate the tissue sodium signal based on differences in ^23^Na relaxation properties in different tissues.^27^ Two different approaches based on the DA‐3DPR sequence were provided: the first used an inversion recovery (IR) preparation pulse to exploit T_1_‐differences of ^23^Na ions, and the second approach was based on ^23^Na multiecho sequences to exploit differences in T_2_*‐relaxation times. However, the relaxation‐weighted ^23^Na signal describes a compartment defined by ^23^Na relaxation properties and does not necessarily correspond to the intra‐ or extracellular space.^28^

Although several different methods for sodium DQ and TQ coherence discrimination have been proposed in the literature, a clear confirmation that intra‐ and extracellular signals can be separated in vivo is still lacking..^29^ MQC from both the intra‐ and extracellular spaces are similar because of the labile macromolecular interactions that result in comparable relaxation properties (T_2_ values) of sodium nuclei in different environments. Therefore, the difference between intra‐ and extracellular sodium signals cannot be established based on relaxation time constant characteristics. This is certainly one of the most important topics on which the sodium MRI community should come to a consensus in the future.

## Image Reconstruction Methods

MR is fundamentally a low SNR imaging method, and the use of nuclei other than protons for imaging purposes may have a considerable impact on the resulting image SNR. The acceleration of image acquisition allows significant improvements in SNR per unit time.

Pioneering work in this field was done by Qian et al,^30^ who demonstrated the advantages of parallel imaging with a TPI trajectory. In computer simulations, these authors tested the TPI‐SENSE (sensitivity encoding) method with an applied acceleration factor of 5.53 and simulations were verified on the proton human head studies. The results showed that parallel sodium imaging may reduce scan time substantially compared to the conventional TPI acquisition (3 minutes vs. 16 minutes) without a substantial loss in image quality.^30^ Several years later, Benkhedah et al investigated feasibility of the adaptive combination reconstruction (ADC) method for multichannel coil array data combining and found that mean SNR may be increased from 8–50% compared to standard sum‐of‐squares (SOS) image reconstruction.^31^ Additionally, an alternative possibility for high‐quality image reconstruction from undersampled datasets is a compressed sensing (CS) method. Gnahm et al recently showed that an anatomically weighted second‐order total variation reconstruction of ^23^Na MRI using prior information from ^1^H MRI (AnaWeTV) provides an increase in image quality due to maintained tissue borders and reduced partial volume effects (PVE).^32^ A method that uses a 3D, dictionary‐learning CS reconstruction algorithm (3D‐DLCS) for the reconstruction of undersampled 3D radial ^23^Na data was presented by Behl et al.^33^ Using the dictionary, it is possible to learn the sparsifying transform with a K‐singular‐value‐decomposition (K‐SVD) algorithm. The same method was shown to be feasible for tissue sodium concentration (TSC) quantification of skeletal muscle.^34^

## Quantitative Sodium MRI

A comprehensive review article about the evolving role of quantitative sodium imaging in medicine was recently published by Thulborn.^12^ Different applications of ^23^Na‐MRI for quantitative analysis, particularly of the musculoskeletal system, were reviewed by Bangerter et al.^35^

In a study published in 2010, Lu et al presented a flow chart for an image reconstruction and tissue sodium quantification (TSC) process that included B_0_, B_1_ mapping, and eddy current corrections, assuming that ^1^H and ^23^Na coils have the same eddy current characteristics.^16^ These authors investigated the effect of B_0_ corrections on TSC measurements and found that a substantial improvement in image quality and quantification accuracy can be achieved by introducing B_0_ corrections in an image postprocessing scheme. The effect of B_1_ inhomogeneity on absolute ^23^Na concentration quantification was further studied by Lommen et al, who proposed a method for simultaneous B_1_ mapping (implemented into the 3D radial projection sequence) and ^23^Na imaging to increase accuracy and to reduce measurement time.^36^ Niesporek et al developed an algorithm for partial volume effect (PVE) corrections and demonstrated a high performance of the method to reduce the discrepancy between the measured and expected sodium concentration value by 11% to a mean PVE‐caused inconsistency of 5.7% after correction.^37^

## RF Systems for Sodium MRI

RF coils are one of the most important elements for high‐quality, high‐SNR sodium MRI. There are a number of challenges connected to RF coil design, first among them the operating frequencies, which have to be low; consequently, this can lead to problems maintaining good coil loading, and thus, directly limits coil sensitivity. The performances of different RF coil designs for sodium imaging of brain and musculoskeletal applications have been extensively discussed in a review article published by Wiggins et al.^10^ The overview of the advantages and disadvantages of various RF coil topologies for sodium body imaging has been reported by Bangerter et al.^11^

Because of the very short T_2_ of sodium, short RF pulses are required to minimize echo time, which drives a highly efficient transmit coil to limit the maximum voltages needed. The benefit of a linear increase in SNR with field strength is diminished by higher specific absorption rates (SARs), which require a lengthening of the RF pulse durations or repetition times.

One of the major demands of clinicians is that the same coil provides proton and sodium imaging within one imaging session. Moreover, to maximize sodium MRI performance, it is important to maximize the uniformity of the main B_0_ static magnetic field. The most efficient and robust way is to calculate the correct shim currents using proton‐based methods to map the B_0_ field and then transfer shim parameters to sodium imaging. This involves the use of double‐tuned, multichannel phased array coils. However, the major issue with dual frequency designs is that they degrade the performance at one or both frequencies (^1^H coil structures have to cope with screening effects as a result of currents induced on the ^23^Na coil part).^10^ Recently, Gast et al developed a ^23^Na‐MRI‐based method for localized B_0_ shimming at 7.0T and showed that an external field inhomogeneity can be reduced up to 77% using this approach.^38^ Many different coil designs, optimized for improved sodium sensitivity, have been developed and reported in the literature recently, suitable for almost all body parts, such as the knee, the breast, and the brain.^10^, ^11^, ^39^, ^40^, ^41^, ^42^, ^43^, ^44^, ^45^

## Sodium Imaging of Cartilage

### Cartilage Repair

Within the last two decades, a large number of cartilage repair surgical techniques were developed, such as bone marrow stimulation techniques (Pridie drilling, microfracturing, MFX), first‐, second‐, and third‐generation cell‐based autologous chondrocyte implantation (ACI), autologous osteochondral transplantation (AOT), and cell‐free implant techniques. A recent review article by Zbyn et al provided an extensive overview of different repair techniques evaluations using ^23^Na‐MRI.^8^

**FIGURE 1:**
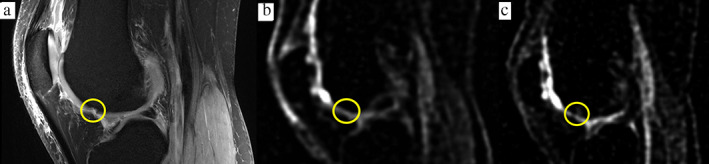
A 39‐year‐old male patient who had a small cartilage defect less than 15% of the cartilage thickness (ICRS grade 2 lesion) in the proximal trochlear region of the lateral femoral condyle. **(a)** A fat‐suppressed proton density (FS‐PD) image in a sagittal orientation shows a lesion with intra‐chondral signal alterations. Sodium ^23^Na‐MRI images generated **(b)** 3 days and **(c)** 4 weeks after the defect.

Proton MRI allows the morphological assessment of the cartilage and cartilage repair tissue and, more recently, biochemical assessment using the glycosaminoglycan (GAG) chemical exchange saturation transfer (CEST) technique. Information about the fine structure or biochemical composition and the quality of the repair tissue is, however, of particular interest, as it has been demonstrated that the composition of the repair tissue may affect the long‐term outcome. In addition to gagCEST, ^23^Na‐MRI is able to assess changes in ion content connected to GAG molecules. The GAGs are negatively charged molecules and belong to the group of the most important constituents of cartilage. GAG is the central point of molecular investigations of the cartilage tissue because it has a significant influence on its function and homeostasis. Furthermore, the GAG content significantly correlates with the biomechanical properties of cartilage, in particular, compressive stiffness.^46^ In articular cartilage, the negatively charged GAGs are counterbalanced by positively charged sodium ions; thus, the sodium concentration can be used as an indirect indicator of the amount of GAG, which, in turn, can be noninvasively assessed using sodium imaging^47^, ^48^ (Fig. 1). For quantitative measurements, phantoms with known sodium concentrations may be placed close to the organ under investigation and may provide absolute tissue sodium concentrations.

The first study that included patients after cartilage repair treatment was published by Trattnig et al in 2010.^49^ Twelve patients were involved in the study and scanned using a 3D‐GRE (gradient echo) sequence with a sodium‐only, birdcage knee coil. Each patient was examined ~56 months after matrix‐associated chondrocyte transplantation (MACT). The normalized values of the sodium normalized signal intensity (NMSI) were significantly lower in transplanted tissue (174 ± 53) than in reference cartilage (267 ± 42) (*P* < 0.001). When the results were compared with dGEMRIC (delayed Gadolinium Enhanced MRI of cartilage), another GAG‐specific method, the authors concluded that sodium MRI allows the differentiation between MACT repair tissue and native cartilage of patients without the need for contrast agent application. For validation of gagCEST imaging as a new method sensitive to changes in GAG content, Schmitt et al compared gagCEST results at 7.0T with sodium MRI in patients with femoral cartilage repair.^50^ Results based on five MFX and seven MACT patients showed a strong correlation between sodium and gagCEST values and demonstrated the feasibility of this method for the cartilage GAG content assessment.

Zbyn et al further investigated the quality of newly developed repair tissue on femoral condyle cartilage after two different repair procedures: bone marrow stimulation (BMS) and MACT.^51^ The NMSI of repaired tissue was significantly lower in patients after BMS than in those who underwent a MACT procedure ((164 ± 31) vs. (210 ± 36)) (*P* = 0.028). However, the properties of the repair tissue, evaluated by the MOCART scoring system based on morphological MR evaluations,^52^ were not significantly different between two groups of patients after BMS and after MACT treatments (*P* = 0.915). The sodium results suggest that a higher GAG content is common for the sophisticated cell‐based MACT technique compared to the more simple BMS technique. This basically means that MACT produces high‐quality repair tissue with a more hyaline‐like composition compared to BMS, which mainly produces fibrous repair tissue with a very low GAG content. Sodium MRI can differentiate between repair tissues with different amounts of GAG, and thus, serve for the noninvasive evaluation of the performance of new cartilage repair techniques.

An article by Chang et al compared sodium MRI results obtained from in 11 patients with and without fluid suppression, after several different procedures of cartilage repair (MFX, MACT, osteochondral grafting, juvenile cartilage implantation). Examinations were done at 7.0T employing a radial UTE sequence, a sodium‐only, birdcage knee coil, and IR sequence with an adiabatic inversion pulse.^53^ The results of the study demonstrated that fluid‐suppressed sodium MRI is more robust compared to standard sequences. In addition, sodium concentration in neighboring cartilage to transplanted tissue was significantly lower than in healthy cartilage tissue. This is in agreement with the findings of in vitro studies, which demonstrated that the amount of viable chondrocytes is lower close to the site of injury.^54^

Cartilage defects can occur also in the ankle joint, mostly after injury or in patients with osteochondritis dissecans. Cartilage repair procedures used in the knee joint are also performed in the ankle joint. MRI of the ankle joint is particularly challenging for a number of reasons. The cartilage lining is curved, highly congruent, and significantly thinner than in the knee joint, with an average thickness of 1.1 ± 0.18 mm for talar cartilage and 1.16 ± 0.14 mm for the cartilage of the distal tibia.^55^ This leads to increasing problems regarding partial volume effects and SNR. The first results of sodium imaging in cartilage repair technique evaluations were promising; therefore, Zbyn et al applied a similar approach to cartilage repair technique evaluations after MFX and MACT in the ankle joint.^48^ After biochemical validation of the sodium imaging protocol using ex vivo measurements of ankle joint cadavers, which demonstrated a strong linear correlation between the NMSI and the histochemically assessed GAG content (*r* = 0.800; *P* < 0.001; R^2^ = 0.639), reference values were obtained for healthy volunteers. Subsequently, patients after MFX and MACT of the talar dome were examined. The repair tissue of both treatment groups exhibited significantly lower corrected signal intensities (cSI) compared to healthy cartilage (MFX, *P* = 0.007; MACT, *P* = 0.008), indicating a lower GAG content than in healthy reference cartilage of the same patients or the healthy controls, but no significant difference was found between both treatment groups, indicating that cartilage composition and the effect on repair seems to be different in the ankle joint compared to the knee joint (*P* = 0.185). No significant difference in cSI was found between healthy cartilage of subjects and patients (*P* = 0.355) as well.

Quantitative TSC imaging of articular cartilage is challenging and its accuracy is limited by several factors, such as the partial volume effect caused by the relatively low spatial resolution, signal loss from T_2short_ decay due to the insufficiently short TE, and T_2_ blurring due to rapid signal decay during the readout. In the future, protocols for TSC quantification that are feasible for clinical applications should be further developed.

## ^23^Na‐MRI in Osteoarthritis (OA)

**FIGURE 2:**
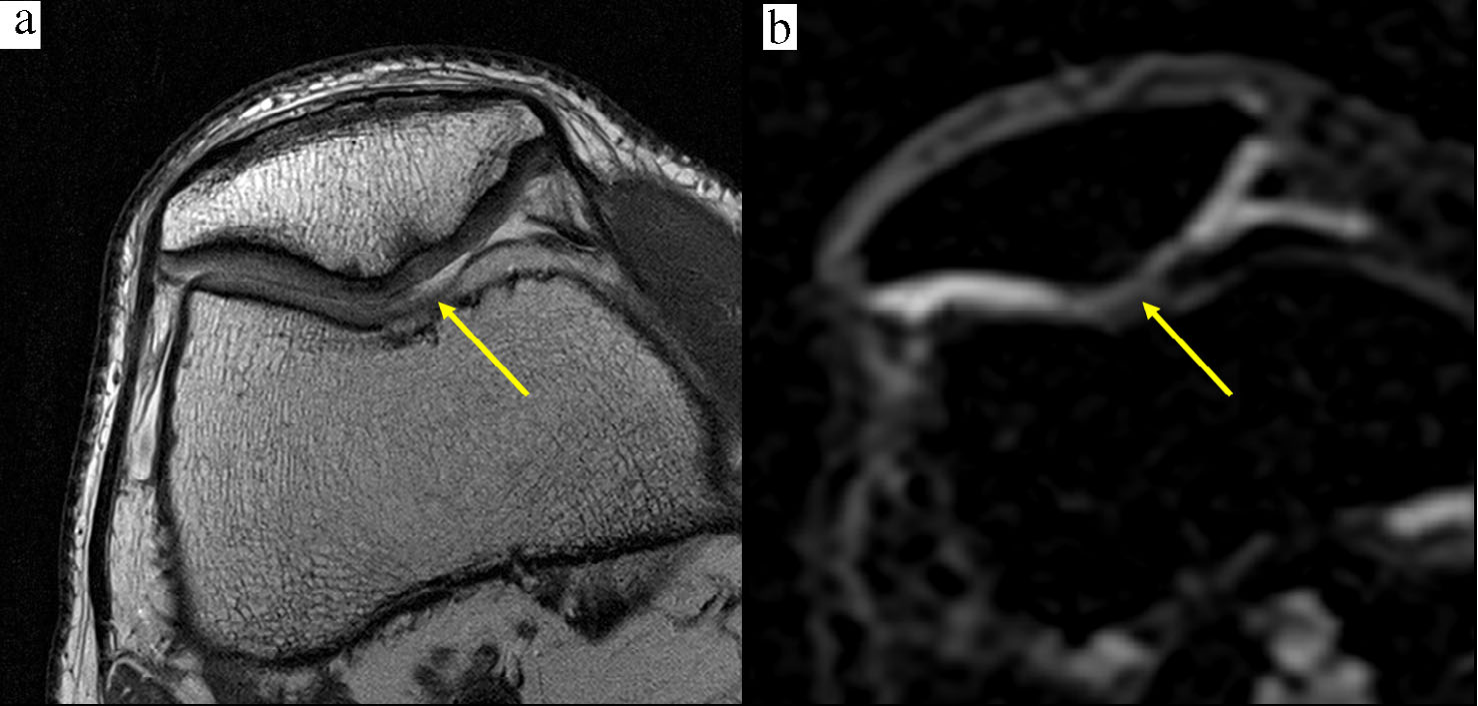
A 50‐year‐old male patient with a lesion in the patellar cartilage in the area crista patellae. **(a)** A proton density sequence with fat suppression (FS‐PD) demonstrates an early stage degeneration of articular cartilage with a minor chondral signal alteration and a minor reduction of cartilage thickness, and a surface that appears intact (ICRS grade 1 lesion). **(b)** Yellow arrow shows the corresponding area on the sodium image.

Proton (^1^H) MRI methods provide different information about the morphology of the knee joint, but the more valuable diagnostic information is that regarding compositional changes in the joint, which often occur before morphological changes appear (Fig. 2). This gives rise to the need for biochemical and quantitative MRI to reveal the early changes in the complex molecular composition of articular cartilage.

After validation in in vitro studies and in studies on animal models,^56^ sodium MRI was employed for the evaluation of OA patients. Wheaton et al performed a study using a surface coil at 4.0T and acquired sodium images with a UTE radial sequence.^57^ These authors measured the knees of nine healthy volunteers without and three patients with symptoms of early OA. The mean sodium content measured in the patellae of the volunteers corresponded to a mean fixed charge density (FCD) measurement of –182 ± 9 mmol/L. These authors generated sodium maps for each OA patient, which illustrated cartilage regions with significantly lower FCD (from –108 to –144 mmol/L) when compared to the FCD of healthy volunteers.

Zbyn et al very recently demonstrated that in vivo ^23^Na‐MRI is a feasible method for the differentiation between low‐grade lesions and normal‐appearing articular cartilage.^58^ Morphological MRI at 3.0T and biochemical ^23^Na‐MRI at 7.0T investigations were performed at four timepoints (baseline, 1 week, 3 months, and 6 months). After every MR session, patients underwent the Knee Injury and Osteoarthritis Outcome Score (KOOS) questionnaire for clinical evaluation. The authors found significantly lower ^23^Na‐cSI values in all lesions than in healthy cartilage tissue at all timepoints (all *P* ≤ 0.002). KOOS scores improved in all subscales at the 3‐ and 6‐month visits, with a significant increase observed only in the quality of life subscale (*P* = 0.004).

The results of all the above‐mentioned publications confirmed that sodium is a feasible and reliable method for the assessment of early OA changes. Findings of the clinical studies support the concept that compositional cartilage changes can develop earlier than progressive morphological changes are detectable. This information may be crucial for early OA discovery and the efficacy of new treatment options in OA.

## ^23^Na‐MRI of the Spine

The intervertebral disk shows a characteristic architecture tailored to its biomechanical purpose. The nucleus pulposus (NP) shows a high concentration of proteoglycans (PG) in the extracellular matrix that consists of large complexes of bound GAGs. Those, in turn, have the essential ability to passively store water due to their negatively charged side chains that attract free‐floating, positively charged ^23^Na ions. Therefore, unlike most other tissues in the human body, cartilage, in general, and the intervertebral disc (IVD), specifically, have the vast majority of ^23^Na ions present in the extracellular volume.^6^

The idea to detect early biochemical changes in the intervertebral disc due to disk degeneration using ^23^Na‐MRI was conceptualized and measured in vivo using a DQF, a DQF‐MA, as well as a TQF sequence by Ooms et al.^59^, ^60^ The group identified age‐related changes in ^23^Na‐MRI of the thoracic and lumbar intervertebral discs. First, the content and the residual quadrupole interaction in the nucleus pulposus and the annulus fibrous differed at different ages. Also, the quadrupole coupling (ωQ) and the relaxation rate of satellite transitions (1/T_2f_) increased with age.

An ex vivo study published by Wang et al used bovine intervertebral discs and measured the PG content using 1,9‐dimethylmethylene blue (DMMB) assays of 28 nucleus pulposus punch samples.^61^ As already shown by Urban et al, a nucleus pulposus without measurable PG content still has a remaining ^23^Na concentration of about 111.54 mmol/L.^62^ Wang et al combined their measurements and the prior work published by Urban et al^62^ and showed a correlation coefficient of 0.71 between the PG content measured by DMMB and the ^23^Na measured by sodium MRI. Simulating the relationship only for a ^23^Na concentration from 150 mmol/L to 350 mmol/L, a linear correlation coefficient of 0.998 was reached for sodium MRI, making it a very potent imaging biomarker for the indirect quantification of GAGs in the intervertebral disk.^61^

Noebauer‐Huhmann et al published an in vivo study confirming that the concept of sodium imaging was a feasible, noninvasive imaging biomarker, and compared it to T_2_ mapping and morphological grading.^63^ As degeneration is also associated with the diminishing synthesis of proteoglycans, sodium imaging might be able to longitudinally depict temporal changes in different disc pathologies.^63^ However, those authors did not find a significant correlation of T_2_ values and ^23^Na imaging. This was expected, since T_2_ mapping is mainly related to water content, collagen fiber content, and organization, while sodium imaging correlates with GAG content. In addition, it is known that natural degradation of the disc lowers the measured T_2_ relaxation times due to the accumulation of lipids and adducts from carbohydrates, which does not affect the ^23^Na imaging measurements. This allows for the assumption that ^23^Na‐MRI can distinguish between nondegenerative, age‐related pathological changes in the extracellular matrix of the nucleus pulposus, potentially making it more sensitive to pathophysiological changes than other quantitative MRI methods.

## Sodium in Tendons

Sodium MRI is a useful imaging modality for Achilles tendon biochemical investigations. The Achilles tendon connects the calcaneus and the calf muscle and must withstand a remarkable load during movement. To facilitate that, the highly organized collagen matrix provides the base for static biomechanical properties with the aid of proteoglycans (~3.5% of dry weight) responsible for dynamic biomechanical properties. In tendinopathy, due to stimulated proteosynthesis, which helps to overcome disaggregation of the microfibrillar bundles, increased proteoglycan concentration has been observed using biochemical assays.^64^ As negatively charged sulfate and carboxyl groups of proteoglycans attract positively charged sodium ions, there is a direct proportion of sodium and proteoglycan concentration in the Achilles tendon. To acquire a ^23^Na signal from the tendon, typically no dedicated coils are used, but sodium knee coils are used instead—they provide enough space for lower leg placement and satisfactory coil loading. Juras et al showed that the sodium signal can serve as a marker for Achilles tendinopathy, as it provides information about elevated sodium concentration in the tendon.^65^ They investigated eight patients with clinical findings of chronic Achilles tendinopathy and scanned them with a 3D‐gradient echo sequence using an in‐plane resolution of 0.89 mm and a total measurement time of about 32 minutes. Mean bulk sodium SNR observed in this study was 4.9 ± 2.1 (a.i.) in healthy control subjects and 9.3 ± 2.3 (a.i.) in patients with Achilles tendinopathy, and these means were statistically significant. Interestingly, these increases were not only local, suggesting that the tendon is completely affected in Achilles tendinopathy. Also, the validation of the sodium signal and sodium content in tendons was investigated in the same study.^65^ Using 15 cadaver samples of Achilles tendons, which were analyzed with regard to the proteoglycan content, the Pearson correlation coefficient between the sodium SI and the proteoglycan content in dry weight was 0.71.

In another study, the increased sodium content in the Achilles tendon, after fluoroquinolone antibiotic therapy, was investigated with ^23^Na‐MRI.^66^ It has been previously observed that, after fluoroquinolone treatment, patients can develop Achilles tendinopathy, including all the typical symptoms, such as acute onset of tendon pain, tenderness, and swelling that affects the function of the tendon and, in the worst case, may lead to tendon tear. Seven healthy male subjects underwent voluntary ciprofloxacin treatment (1000 mg/day in two doses: 500 mg in the morning and 500 mg in the evening for 10 days) and were scanned with ^23^Na MRI at three timepoints: baseline; at 10 days; and at 5 months after the treatment. Here, the variable echo‐time (vTE) sequence adapted to x‐nuclei capabilities was used with an echo of 2.45 msec and a total acquisition time of 15 minutes. Despite the fact that there were no morphologically detectable changes in these volunteers after the treatment, the NMSI decreased by almost 25% (from 130 ± 8 to 98 ± 5 a.u.) and then reached 116 ± 10 a.u. after 5 months. The results provide evidence that sodium MRI is a technique robust and sensitive enough to detect the changes in the sodium content in the Achilles tendon, which could be associated with altered proteoglycan content and may pose the tendon to risk for tendinopathy and tear.

## Muscle

Recent studies demonstrated that ^23^Na‐MRI of a lower leg muscle can be a useful imaging modality for the detection of sodium content changes during exercise or disease.^67^, ^68^ The sodium concentration increase or decrease in the body manifests as a condition known as hypernatremia or hyponatremia, which may be induced by a hormonal imbalance or related diseases, such as diabetes mellitus, hypertension, and acute heart failure. ^23^Na concentrations alterations in calf muscle, however, may be closely related to developed pathologies of the muscle tissue, such as channelopathy and muscular dystrophy. Several studies confirmed the reproducibility and repeatability of quantitative sodium imaging in the lower leg muscle, which is considered a basic condition for an accurate evaluation of several different muscle pathologies in patients.^69^, ^70^

### Sodium Evaluations in Diabetic Patients

Na^+^/K^+^‐ATPase activity is decreased in patients suffering from diabetes. A sufficiently high concentration of hormone insulin in the blood will directly enhance sodium/potassium pump activity in muscle, kidney, liver, etc.^71^ Chang et al evaluated the signal intensity (SI) of sodium pre‐ and postexercise. The sodium signal was assessed in patients with diabetes and in healthy subjects, in all three compartments of the calf muscle: tibialis anterior (TA), soleus (S), and the gastrocnemius (G) muscles. It was found that the sodium signal intensity in the S and G immediately increased significantly after exercise for both diabetic patients and healthy subjects. However, the signal intensity decrease to baseline was slower in diabetics. An explanation for this can be supported by the fact that, in patients with diabetes mellitus, muscle function is reduced and accompanied by impaired functioning of the Na^+^ /K^+^ pump.

### Muscular Channelopathy and Muscular Dystrophy

Muscular channelopathies are a group of nondystrophic myopathies, which are caused by gene mutations that result in a malfunction of the ionic channels of the muscle. Nagel et al, as a clinical model, chose patients with hypokalemic periodic paralysis (hypoPP) and paramyotonia congenita (PC).^72^ The purpose of the study was to investigate which of three different sequences: ^23^Na‐TSC; T_1_‐weighted sodium imaging (^23^Na‐T_1_); or ^23^Na‐IR would provide the strongest weighting toward intracellular sodium. All three sequences demonstrated significantly higher signal intensities in hypoPP compared to those in PC patients and healthy subjects. However, after inducing a provocation in PC patients, a significant (*P* = 0.007) increase (>20%) in the muscular ^23^Na‐IR signal and a corresponding decrease of muscle strength was detected. These results provide strong evidence that ^23^Na‐IR substantially advances weighting toward intracellular sodium and enables an improved evaluation of pathophysiological changes of muscles in patients suffering from rare diseases.

**FIGURE 3:**
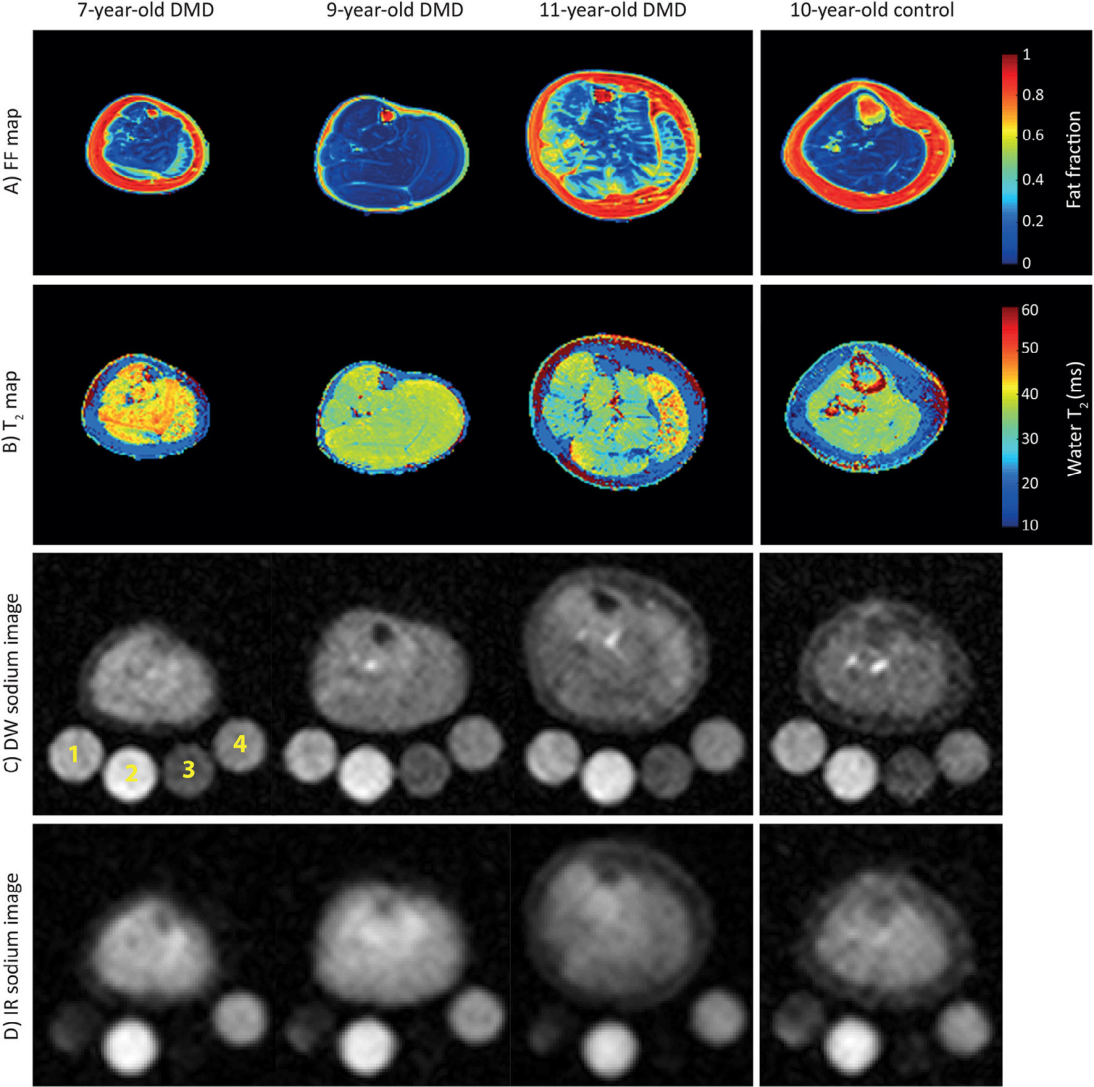
Illustration of **(a)** fat fraction maps, **(b)** water T_2_ maps, **(c)** DW ^23^Na images, and **(d)** IR ^23^Na images in the leg of 7‐year‐old, 9‐year‐old, and 11‐year‐old DMD patients, as well as a 10‐year‐old control. For ^23^Na MRI, four reference tubes were used (1, 40  mM NaCl; 2, 40 mM NaCl, and 5% agarose gel; 3, 20 mM NaCl; 4, 20 mM NaCl, and 5% agarose gel). The leg muscles of DMD patients showed generally elevated FF, water T_2_, and sodium signals compared with age‐matched controls. While the 7‐year‐old DMD patient exhibited slightly elevated FF (mean FF 0.08 ± 0.04), the FF was much higher in the muscles of the 11‐year‐old DMD patient (mean FF 0.19 ± 0.1), who was not able to walk more than 10 m without human assistance. Increased total sodium and intracellular‐weighted sodium signals are also visible in the dystrophic muscle tissue with normal T_2_ and FF values (figure reproduced from Ref. 73 with permission from Wiley).

In contrast to skeletal muscle channelopathies, which are rare, inherited childhood‐onset disorders, myotonic dystrophy (DM) is the most common form of muscular dystrophy that begins in adulthood. A special severe type of DM, investigated with ^23^Na‐MRI, is Duchenne's muscular dystrophy (DMD) that is usually diagnosed in childhood. DMD is caused by a mutation in the dystrophin gene, which leads to progressive muscle weakness and destruction and is associated with ion homeostasis dysregulation and chronic inflammation.^73^ Gerhalter et al studied 13 patients with DMD and found that TSC (26.0 ± 1.3 mM, *P* < 0.05) and ICS (0.69 ± 0.05 a.u., *P* < 0.05) were elevated in DMD compared to healthy controls (16.5 ± 1.3 mM and 0.47 ± 0.04 a.u.). The ICS was frequently abnormal in DMD compared to heathy controls, and was present even in the absence of fatty degenerative changes and water T_2_ increases^73^ (Fig. 3). One explanation for the observed increased ICS and TSC values despite normal water T_2_ values might be that sodium MRI is more sensitive in the detection of dystrophic changes. Since there were no significant changes in the ICS/TSC ratio between the healthy and dystrophic muscle, this observation could be compatible with two concurrent phenomena: an increase in the intracellular sodium and an increase of the extracellular volume fraction.

### Hypertension

Quantitative ^23^Na‐MRI in muscle may be used to assess an increase in body sodium content due to high blood pressure (HBP). Kopp et al found a 29% increase in ^23^Na content in patients with aldosteronism associated with hypertension compared to healthy subjects.^74^, ^75^ Although the authors stated that the achieved resolution (3 × 3 × 30) mm^3^ was adequate, they considered it as unsatisfactory for the skin sodium concentration evaluations. However, the authors' aim was to test the basic hypothesis about Na^+^ storage in muscle, and particularly, in skin, without apparent accompanying fluid retention. Linz et al developed a dedicated, two‐channel transceiver RF coil array for skin measurements and performed sodium imaging in humans with a gradient echo sequence and 0.9 × 0.9 × 30 mm^3^ resolution at 7.0T.^76^ Skin sodium concentration results showed a discrepancy between the Na^+^ content obtained in vivo at 3.0T vs. 7.0T. The inconsistency could be the result of the higher spatial resolution at 7.0T, which may compensate for the PVE that occurred at the lower field strength. Submillimeter image resolution, achievable on ultrahigh MR systems, should provide a better understanding of the physiological processes related to HBP in the future.

## Cardiac ^23^Na MRI

### Technical Considerations

Sodium is an essential ion for myocyte function and integrity. It plays an important role in balancing osmotic pressure through the sodium–potassium pump. The concentration of extracellular sodium is ~10 times higher than that of the intracellular concentration to provide proper muscle excitation, conduction, and contraction. The pathological change in TSC and the impairment of sodium flux often occurs in hypertrophic myopathies, ischemic cardiac diseases, and infarction. Hence, ^23^Na‐MRI provides a valuable and powerful imaging tool for the detection of sodium concentration in heart muscle in vivo. However, similar to other sodium MR applications, cardiac sodium MR faces many issues related to physical and technical obstacles. An article published by Bottomley provided a critical review of the properties, methods, and potential clinical applications of ^23^Na‐MRI in the human heart.^7^

Cardiac ^23^Na‐MRI studies may be performed on MRI scanners from 1.5–7.0T with a spatial resolution in the range of 100–1000 mm^3^, typically 160 mm^3^ at 3.0T in a 10‐minute measurement time.^7^, ^77^ The relatively short T_1_ relaxation in heart muscle allows for further scan time shortening by increasing the number of signal averages. The ^23^Na signal in heart tissue usually exhibits a bicomponent decay, with an ~40% fast component (T_2f_, ranging from 0.5–4 msec) and a 60% slow component (T_2s_, ranging from 12–32 msec).^78^, ^79^, ^80^ With conventional MR techniques using an echo time (TE) longer than 3 msec, the majority of T_2f_ is lost; however, using short‐ and ultrashort MR sequences, the TSC can be measured quite precisely and the values match the assay.^78^

For clinical applications, it would be desirable to separate intracellular sodium, typically 10–15 mM and extracellular sodium, typically 135–150 mM. The theory that attributes intra‐ and extracellular sodium to T_2f_ and T_2s_ is controversially discussed in the literature, as there is a lack of solid evidence to prove this theory. There is even some evidence against this theory, suggesting that, from an NMR point of view, the ^23^Na ion is similar inside the cell and outside the cell.^81^ The sodium signal is a relative value determined by a combination of many factors, such as T_1_, T_2_, B_1,_, and B_0_ field homogeneity, and is linearly correlated with sodium concentration in heart muscle tissue. It is also possible to measure absolute TSC in the heart using reference tubes with a known sodium concentration. These reference tubes are scanned either separately with the same protocol or in the field of view of an actual ^23^ Na‐MRI measurement of the heart, taking into account the coil sensitivity. There have also been attempts to use an internal reference that does not change between subjects, eg, myocardial and ventricular blood ratio; however, a direct relation to TSC has not, as yet, been validated.^82^ To acquire the best possible ^23^Na signal, dedicated transmit/receive resonators should be used. Some of these are commercially available,^83^, ^84^ but many research groups rely on custom‐made, dedicated cardiac coils.^7^, ^64^, ^85^ Boehmert et al built a dedicated 4/4 channel ^1^H/^23^Na coil with high B_1_^+^ homogeneity and penetration depth to investigate the cardiorenal syndrome.^86^ The complexity of the dedicated resonators grows with the field strength. Typically, a surface coil design is preferred, with the diameter approximately equal to the depth of interest, which is 10–15 cm. Another option for increasing ^23^Na SNR is phased array surface coils; however, multichannel ^23^Na‐MRI is required, which is available on only a small number of MR scanners installed worldwide.^85^, ^87^

To acquire the maximum sodium signal from the heart and allow for TSC quantification, MR sequences with short TEs are necessary.^64^, ^77^, ^79^ Typically, 3D projection reconstruction techniques are used, with a hard excitation pulse followed by a constant gradient. To acquire an optimal SNR, the projection increment is 111°, referred to as the “golden ratio.”^79^, ^88^ Other possible trajectories are twisted projection imaging^16^ and spiral imaging.^89^ As hard pulses are very sensitive to B_1_ inhomogeneity, adiabatic pulses are alternatively used to provide a B_1_‐independent excitation. Cardiac motion is also a pitfall for ^23^Na‐MRI sequences, and electrocardiography (ECG) gating, as often used in ^1^H cardiac imaging, is not helpful because the advantage of the short T_*1*_ of ^23^Na would be lost. Therefore, either only motion‐quiet frames are acquired^84^, ^90^ or retrospective ECG gating is used.^90^, ^91^

### Preclinical and Clinical Studies

Animal models provide an important step toward understanding the physiology and pathology of sodium in the heart, although the small structures present considerable technical challenges.^78^, ^92^, ^93^, ^94^ However, it is possible to compensate for the lower SNR by using small‐bore, ultrahigh‐field MR scanners. Neuberger et al used a 17.6T animal scanner to acquire high‐resolution sodium images of the mouse heart.^93^ The sodium SNR in the left ventricle, the right ventricle, the left ventricular free wall, and the septum was found to be 9.2 ± 2.3, 8.3 ± 2.06, 3.8 ± 1.0, and 5.6 ± 1.4, respectively. In another study, sodium imaging and spectroscopy was investigated for use as a potential marker with which to assess viability after low‐flow ischemia, using a rat heart model.^92^ Intracellular sodium image intensity increased significantly during ischemia of the left side, whereas that of the right side remained unchanged; however, total sodium image intensity remained unchanged in both sides of the heart.

**FIGURE 4:**
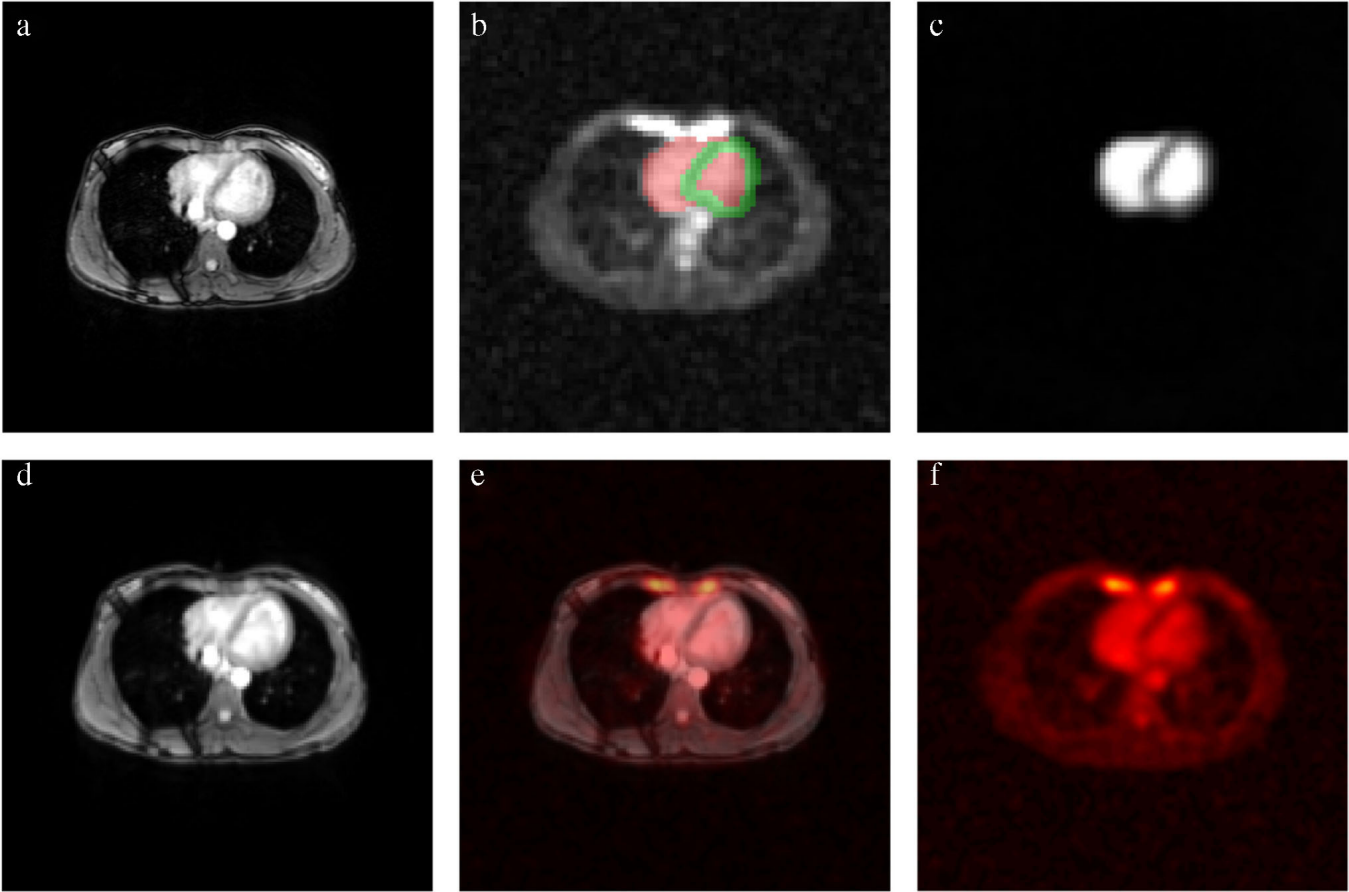
An example of proton and sodium images with segmentation masks used for quantification and postprocessing. **(a)** ^1^H MRI image (with navigator stripes) before registration. **(b)** ^23^Na MRI with corresponding masks (red = blood mask, green = myocardial mask) based on the ^1^H image. **(c)** Simulated ^23^Na MRI of the heart based on ^1^H masks. **(d)** ^1^H MRI image after registration. **(e)** Registered ^1^H image with cardiac ^23^Na MRI as an overlay. **(f)** Cardiac ^23^Na MRI (figure adapted from Ref. 96 and reproduced with permission from Wiley).

^23^Na‐MRI offers a tool with which to study myocardial ion homeostasis in vivo and can be used for different areas of cardiovascular disease. Most of the clinical studies are related to the investigation of sodium levels in myocardial infarction (MI). In a feasibility study by Standstede et al, the elevated sodium signal was observed in the myocardium affected by acute infarction (60.6 ± 21.6) compared to healthy controls (37.2 ± 12.8); however, no statistically significant increase was measured in infarcted myocardium in the subacute and the chronic groups.^95^ To acquire the sodium signal from the heart, an ECG‐triggered, 3D, spoiled gradient‐echo (fast low‐angle shot) sequence with a TE of 3.1 msec was used. In a follow‐up study, the same authors monitored 12 patients on days 4, 14, and 90 after infarction and found an increase in the sodium signal of 39 ± 18, 31 ± 17, and 28 ± 13 [%], respectively, suggesting that ^23^Na‐MRI may be an applicable method for imaging nonviable myocardium in vivo.^84^ Absolute TSC was measured in 20 patients with a history of prior MI using a 150 mmol/L Na^+^ concentration reference and a coregistration to ^1^H images acquired with a contrast agent.^90^ Although the TSC was elevated by ~30% in patients compared to healthy controls, TSC does not appear to be linked to infarct age or size or to global ventricular function. Quantitative sodium MRI is heavily influenced by cardiac and respiratory motion, PVEs, and inhomogeneity of the static magnetic field, as well as of the transmit and receive field. Lott et al showed that a thorough correction for these influences provides more accurate TSC results from the heart, albeit somewhat lower compared to uncorrected values (Fig. 4).^96^ Christa et al measured increased myocardial sodium SI in Conn's syndrome, which is manifested by hyperaldosteronemia that results in an alteration of sodium and potassium levels.^97^ They used a 3D gradient echo sequence with a TR of 100 msec; a TE of 2.01 msec; flip angle 90°; field of view (FOV) 500 × 500 × 200 mm^3^; which provided an acquisition matrix of 128 × 128 × 10, with a resolution of 3.9 × 3.9 × 20 mm^3^; and eight signal averages; resulting in a total acquisition time of 17 minutes. Patients with Conn's syndrome exhibited significantly higher relative sodium signal intensities in the myocardium compared to healthy controls (0.31, ranging from 0.26–0.34) vs. 0.24 (ranging from 0.20–0.27). The results suggest that the myocardium is, along with skeletal muscle and skin, another possible sodium storage site, and ^23^Na MRI can be used to monitor patients who are undergoing treatment for an aldosterone excess.

## ^23^Na ‐MRI in Kidney Function Evaluation

The most important role of the kidney is to maintain the overall fluid balance in the body. Renal function is determined by a proper regulation of extracellular sodium in the kidney, established by a sodium concentration gradient from the cortex to the medulla. The malfunction of the sodium concentration gradient may be caused by several different renal diseases, such as nephropathy, acute kidney failure, or irregular kidney function after transplantation.

A study by Haneder et al demonstrated the feasibility of in vivo ^23^Na imaging of human kidneys on a whole‐body, ultrahigh‐field MR system at 7.0T. Heathy subjects were examined using a 3D Cartesian spoiled gradient echo sequence with a variable echo time scheme with a nominal in‐plane resolution of 4 × 4 mm^2^ and a slice thickness of 5 mm.^98^ These authors confirmed the validity of the concept of increasing the ^23^Na SNR or ^23^Na concentration from the renal cortex in the direction of the medullary pyramid. This concentration change is called the corticomedullary ^23^Na gradient and it is highly important for proper kidney function evaluation and for the detection of abnormalities.

## Breast Tumors

**FIGURE 5:**
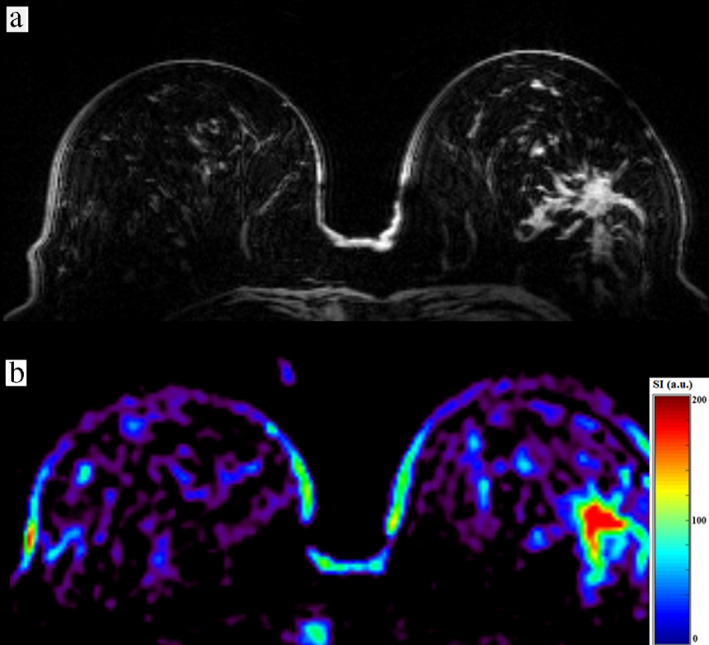
A 60‐year‐old female patient with invasive ductal carcinoma (IDC) and a grade 3 proliferation (G3) in the lateral part of the left breast. **(a)** DIXON water image shows a heterogeneous lesion with irregular margins typical of malignant tumors, and **(b)** a corresponding color‐coded bilateral ^23^Na image corrected for coil sensitivity and obtained with a 3D radial projection sequence (DA‐3DPR).

Ultrasound (US) and mammography are the most‐used imaging modalities for breast lesion detection and characterization. However, the diagnostic accuracy of breast sonography examinations is low, and thus, US is usually recommended as a complementary technique to mammography or other imaging techniques. Mammography, however, is primarily advised for patients over 50 with less dense breast tissue. Low sensitivity and specificity, as well as accompanying ionizing radiation, are strong limitations of this imaging method. The development of noninvasive biochemical MRI techniques is, therefore, necessary, especially those that can define predictive biomarkers for breast cancer diagnosis and characterization with high sensitivity and specificity. One of those techniques that could provide supplementary information to conventional MRI, such as contrast‐based imaging techniques, is ^23^Na‐MRI, which is sensitive to sodium concentration changes in tissue as a reliable biomarker for cell viability and function. Thus, ^23^Na‐MRI may increase overall diagnostic accuracy and contribute to other established imaging methods for the monitoring of treatment efficacy. The technique has been shown to be feasible for the differentiation between malignant and healthy breast tissue and may prevent false‐positive and false‐negative findings in patients at high risk for malignancy (Fig. 5). Zaric et al recently showed that the increased field strength provides the increased sensitivity necessary to achieve an acceptable spatial resolution for the metabolic interpretation of the tissue under investigation.^99^ These authors showed that the low ^23^Na content in healthy glandular breast tissue can be assessed using improved imaging techniques and hardware at 7.0T. The quantitative sodium MRI was performed using an AWSOS sequence. The measurement protocol was optimized, and sodium images with a 1.5 mm in‐plane resolution were acquired in ~16 minutes. It was shown that TSC in carcinomas is 28% and 49% higher compared to benign tumors and healthy glandular tissue, respectively. Lachner et al proposed an advanced CS reconstruction algorithm for radial ^23^Na multicoil data, applied to simulated and measured breast datasets.^100^ For data acquisition, a DA‐3DPR pulse sequence, which enables more efficient *k‐*space sampling, can be used, as well as multicoil arrays that can further enhance the SNR. For the first time, CS was used for undersampled breast sodium data reconstruction. It was demonstrated that CS can be successfully implemented to reduce acquisition time and enhance image quality. Although CS reconstruction could utilize a conventional total variation (TV) denoising technique, it was shown that not only artifacts and noise are diminished, but also small‐tissue structures. As a possible solution, the authors proposed a method that adapts the TV using anatomical weighting factors, which represent known tissue boundaries (AnaWeTV). After inclusion of prior information from proton (^1^H) MRI into the CS reconstruction, image quality was further increased due to preserved tissue boundaries and PV effects. Since proton and sodium images are highly correlated, these weighting factors could be obtained from a high‐resolution ^1^H MR image and then transferred to ^23^Na images. Applied like this, CS reconstruction simultaneously maintained known tissue boundaries and reduced image artifacts. The method still has to be applied and validated in patients with breast lesions.

## Brain

The first clinical NMR images of cerebral sodium distribution in normal volunteers and in patients with a variety of pathological lesions were shown by Hilal et al in 1985.^101^ Subsequently, many different studies explored the potential of using sodium MRI as a noninvasive imaging modality for biochemical investigations of brain tumors, stroke, and neurological disorders. The potential of a combination of sodium imaging with other imaging modalities, such as positron emission tomography (PET) in metabolic change studies connected with human brain pathologies, have been discussed in a review article by Shah et al.^9^ High reproducibility and repeatability of cerebral ^23^Na‐MRI was recently reported by Meyer et al.^102^

### Brain Tumors

Two main histological properties of malignant tumors are increased angiogenesis and cell proliferation. Unregulated Na^+^/H^+^ exchange kinetics and altered Na^+^/K^+^‐ATPase activity may increase the number of cells, which then will lead to tumor genesis and growth. The amount of sodium inside the cell will rise and may be considered a biomarker of tumor malignancy.^103^ One of the first ^23^Na‐MRI studies included 20 patients with brain gliomas, which reported elevated TSC levels for both tumors and surrounding tissues.^104^, ^105^ However, the changes in TSC in a tissue of interest may provide low specificity information on the origin of sodium signal changes and their connection to tumor malignancy.^106^ Intracellular sodium signal that originates from Na^+^ ions with restricted mobility may be distinguished from extracellular sodium signal utilizing TQF sequences. There is currently a controversial discussion within the sodium MRI community regarding the last statement. According to Burstein et al, the intracellular sodium signal cannot be separated from extracellular sodium in human tissue.^29^ The authors explain that, based on chemical kinetics principles, sodium ions in biological systems are not in a “bound” or a “mobile” state due to the fast relaxation rate constants they have.

To date, there is a limited number of publications that have dealt with an application of TQF ^23^Na imaging in patients with brain tumors. Initial reports have suggested a promising role for TQF ^23^Na‐MRI in discriminating vital parts of tumor, with high cell proliferation from areas of tumor necrosis.^24^, ^107^ One method allows simultaneous acquisition of TSC‐weighted, as well as TQF images (SISTINA), utilizing a sequence that interleaves an ultrashort TE, radial projection readout into the three‐pulse, triple‐quantum preparation, and was developed by Fiege et al.^107^ A drawback of the SISTINA method was limited SNR and resolution; tumor images could not be analyzed without a proton reference image. Significant improvements of this technical issue can be achieved with improved readouts, such as DA‐3DPR or TPI sequences. Based on the DA‐3DPR sequence, Nagel et al earlier proposed a relaxation‐weighted (RW) ^23^Na‐MRI and applied it in patients with brain carcinomas. The results showed that RW ^23^Na imaging allows excellent differentiation between grade I–III and grade IV gliomas.^28^ Neto et al recently performed a study with eight brain carcinoma patients before surgical, chemo, or radiation therapy treatment. Patients were scanned at 3.0T using a custom‐made double‐tuned (^23^Na/^1^H) head coil and a FLORET sequence, with and without fluid suppression by inversion recovery (IR). The authors generated maps of pseudo‐intracellular sodium concentration (C_1_), pseudo‐extracellular volume fraction (α_2_), apparent intracellular sodium concentration (aISC), and apparent total sodium concentration (aTSC). These parameters were significantly elevated in the normal‐appearing putamen compared to NAWM. Analysis of all solid tumors demonstrated a significant increase of aTSC and α_2_, and a significant decrease of aISC when compared with NAWM.^108^ In a study published by Biller et al, 34 patients with brain tumors were examined with ^23^Na‐MRI, performed using a 7.0T MR system and a double‐tuned (^1^H/^23^Na) quadrature birdcage head coil, and the DA‐3DPR technique.^109^ The results demonstrated that the initial sodium signal, measured in a brain tumor patient without any previous treatment, is a valuable predictor of isocitrate dehydrogenase (IDH) mutation status and tumor progression. The study confirmed a great potential for ^23^Na‐MRI for an improved and individualized approach in neuro‐oncology.

### Ischemic Stroke

**FIGURE 6:**
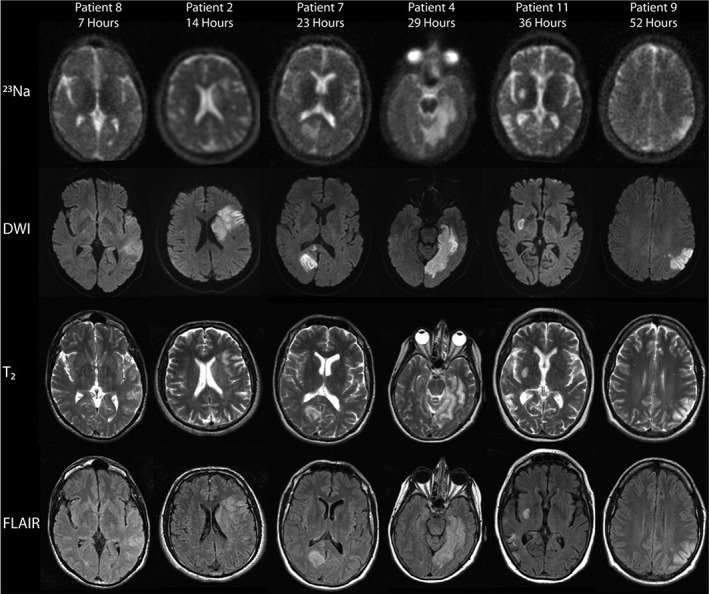
Representative sodium (^23^Na), diffusion‐weighted (DWI), T_2_‐weighted, and fluid‐attenuated inversion recovery (FLAIR) images from patients 7 to 52 hours after ischemic stroke onset. Areas of sodium signal intensity increase correspond to the lesions identified on DWI. The sodium signal intensity in the areas of ischemia qualitatively increased with time (figure reproduced from Ref. 112 with permission from Wiley).

Changes in intracellular ^23^Na concentration are known to occur shortly after an ischemic insult due to impaired functioning of Na^+^/K^+^‐ATPase, which is responsible for human homeostasis. The role of ^23^Na‐MRI in identifying patients suitable for therapeutic intervention immediately after stroke may be crucial (Fig. 6).^16^, ^110^, ^111^, ^112^

### Sodium MRI in Neurological Disorders

Multiple sclerosis (MS) is an inflammatory demyelinating disease that causes the development of focal and diffuse lesions in white matter (WM) and gray matter (GM). Chronically demyelinated MS lesions are accompanied by a substantially reduced axonal Na^+^/K^+^‐ATPase expression.^113^ In vivo MRI studies using ^23^Na imaging showed increased brain TSC in patients with MS. Inglese et al performed a sodium MRI study at 3.0T, including patients with advanced relapsing–remitting MS, and applying a 3D radial gradient‐echo UTE sequence.^114^ These authors quantified absolute TSC in a patient's lesions and several brain regions with normal‐appearing white and gray matter (NAWM and NAGM). The same measurement was performed in corresponding areas in controls. In MS patients, TSCs were higher in MS lesions compared to areas of NAWM. Also, TSC measured in NAWM were also significantly higher than those in corresponding WM regions in healthy controls. Further studies confirmed these findings.^115^, ^116^, ^117^, ^118^ In order to obtain more specific information, Petracca et al used a method that combined SQ and TQF MRI, to quantify TSC and the intracellular sodium molar fraction (ISMF) and then derived ISC and ISVF, an indirect measure of ESC. The results generated from 19 relapsing–remitting MS patients and 17 heathy controls showed that global TSC and ISC evaluated in GM were higher, while GM and WM ISVF (indirect measure of extracellular sodium concentration) were lower in patients compared with healthy controls.^119^ In 11 patients with acute MS lesions, ^1^H and ^23^Na‐MRI examinations were performed at 3.0T.^120^ Initial examinations showed that contrast‐enhancing lesions had high TSC, while, 4 weeks later, MRI TSC in these lesions was reduced. The authors of the study concluded that tissue structure is relatively preserved in the early stage of lesion progression. Stobbe et al investigated potential errors in TSC quantification in patients with primary progressive MS disease, often characterized by small lesions.^121^ The authors found that signal from volumes‐of‐interest (VOIs) in large spheres (~10 cm^3^) was 20% higher than expected. In smaller VOIs (~0.35 cm^3^), the ^23^Na signal was even more underestimated (40–60%). This may be one of the most critical limitations of low‐resolution quantitative methods applied in evaluation of small lesions. A case–control study that included patients who suffered migraines was recently published by Meyer et al.^122^ The results showed a significantly higher sodium concentration in cerebrospinal fluid (CSF) in migraine and tension‐type headache (TTH) patients compared with healthy controls (*P* = 0.007, *P* < 0.001, respectively). Alzheimer's disease (AD) is a chronic neurodegenerative disease accompanied by alterations of the sodium levels in brain due to cell death or loss of functional characteristics. The pathophysiological changes in a brain affected by AD can be measured with sodium MRI and may provide valuable additional information for early discovery of the disease. A small study of AD patients (*n* = 5) reported a 7.5% brain TSC increase with an inverse correlation to the volume of the hippocampus.^123^ Another possible application for ^23^Na‐MRI is in Huntington's disease diagnosis. In patients with this neurological condition, the highest TSC was found in the caudate nucleus, which correlated with GM atrophy.^124^ In a group of healthy subjects, neuroglial‐vascular mechanisms were studied by dynamic sodium imaging using a triple‐echo, 3D‐center‐out radial sequence at 7.0T. These authors demonstrated an activation in the left central regions, the supplementary motor areas, and the left cerebellum, manifested as an increase of the sodium signal at an ultrashort TE and a decrease of the signal at a long TE.^125^ The presumption of the existence of “restricted” and “mobile” sodium ions and differences in the T_2_ relaxation times of sodium nuclei in the intra‐ and extracellular space is, however, still controversial.^29^

## Conclusion

The great potential of ^23^Na‐MRI has been extensively demonstrated. Several hundred publications have presented methodological and feasibility studies showing that ^23^Na‐MRI can serve as a reliable imaging tool for the diagnosis and treatment monitoring of many diseases. The future task of the scientific community is to continue to work on technical improvements of the technique to enable ^23^Na‐MRI to become an approved imaging modality for biochemical clinical investigations and to provide a better understanding of different pathophysiological conditions.
